# Sticky small target: an effective sampling tool for tsetse fly *Glossina fuscipes fuscipes* Newstead, 1910

**DOI:** 10.1186/s13071-018-2840-6

**Published:** 2018-04-25

**Authors:** Njelembo J. Mbewe, Rajinder K. Saini, Baldwyn Torto, Janet Irungu, Abdullahi A. Yusuf, Christian W. W. Pirk

**Affiliations:** 1 International Centre of Insect Physiology and Ecology, P.O Box 30772-00100, Nairobi, Kenya; 2 Department of Zoology and Entomology, University of Pretoria, Private Bag X20, Hatfield, Pretoria, 028 South Africa; 3 Pestinix, International Pest & Vector Control Specialists, P.O. Box 702-00621, Nairobi, Kenya

**Keywords:** Riverine tsetse flies, Small targets, Sampling, Behaviour, Density, Surveillance

## Abstract

**Background:**

Small targets comprising panels of blue and insecticide-treated black netting material each 0.25 × 0.25 m have been shown to attract and kill *Glossina fuscipes fuscipes* Newstead, 1910 (Diptera: Glossinidae) thereby reducing its population density by over 90% in field trials. However, their attractive ability has not been fully exploited for sampling purposes. Therefore, in this study we assessed the effectiveness of using sticky small targets as sampling tools for *G. f. fuscipes* in western Kenya. We also determined the influence of colour on the landing response of female and male flies on sticky small targets.

**Methods:**

Using a series of randomised block experiments, the numbers of tsetse flies caught with sticky small targets were compared with those caught with biconical traps. A negative binomial regression was used to model fly catches. Odds ratios as measures of association between the landing response on the blue or black panel of the sticky small target and the sex of flies were obtained from a multiple logistic regression.

**Results:**

The results showed that sticky small targets caught 13.5 and 3.6 times more female and male tsetse flies than biconical traps (*Z* = 9.551, *P* < 0.0001 and *Z* = 5.978, *P* < 0.0001, respectively). Females had a 1.7 times likelihood of landing on the black panel than males (*Z* = 2.25, *P* = 0.025).

**Conclusion:**

This study suggests that sticky small targets are an effective sampling tool for *G. f. fuscipes.* Therefore, we recommend the use of sticky small targets as an alternative to biconical traps for observational and experimental investigations of *G. f. fuscipes.*

## Background

Human African trypanosomiasis (HAT), also referred to as sleeping sickness is transmitted by tsetse flies harbouring the mature protozoan parasites, *Trypanosoma brucei rhodesiense* and *T. brucei gambiense*, as they take a human blood meal causing Rhodesian and Gambian HAT, respectively [1, 2]. Tsetse flies from the palpalis group which occupy riverine habitats have been implicated in the transmission of over 90% of HAT cases caused by *T. brucei gambiense* in central and west Africa [3, 4]. So far, there is no vaccine and the main intervention for Gambian HAT (gHAT) is the use of case detection and treatment programmes [4]. The method aims to clear the parasite in a substantial proportion of the human population so that even when bitten, the vector will not pick up any trypanosomes for further transmission [1]. However, infected flies can still transmit new gHAT infections to humans [4]. Further, the chronic nature of gHAT allows for silent carriers harbouring low parasite levels undetectable by conventional diagnostic methods to sustain transmission foci [5–7]. Thus, interventions that target the vector further reduce the risk of new infections from occurring [4, 8]. For tsetse fly control, targets are simple, cheaper and easier to maintain than traps [9]. Insecticide-treated targets usually greater or equal to 1.0 × 1.0 m in size are effective control tools for the savannah tsetse fly, *G. pallidipes* Austen and *G. morsitans morsitans* Westwood [10]. Additionally, behavioural studies that aimed at developing cost effective targets for the main vectors of gHAT, *G. fuscipes* subspecies and other riverine species, show that even after reducing the target size 16 times, the alighting response was about 40–55% on the small target, which is comparable to large targets [9]. Further studies found that a small target with a black cloth panel and netting material each 0.25 × 0.25 m in dimensions caught more than twice the number of flies than the biconical trap, and replacing the black with a blue cloth panel having the same dimensions slightly increased the catches [9]. The small targets are highly efficient in reducing tsetse fly densities (by over 90%) and easier and cheaper to deploy than large targets [4, 9, 11]. Apart from being exploited for killing the tsetse fly, small targets could also be exploited for sampling purposes of *G. f. fuscipes* and other riverine species.

Some ecological aspects critical for control of tsetse flies, especially riverine species, include fly movement and spatial occupation within their restricted habitat and can only be studied with adequate sampling techniques [8]. Tsetse fly sampling during studies in the form of observations and experiments are important for planning and monitoring tsetse fly control interventions [8]. In the last four decades, traps that attract and guide tsetse flies into a non-return cage have been developed to serve as sampling or control tools [12]. Although traps have biases and interpretation of catches should be done taking into account these biases; the higher proportion of females to males is close to the natural sex ratio of the tsetse fly population [8, 13, 14].

Sticky panel traps have been mainly used to hold tsetse flies that are killed or stunned by other sampling tools, such as electric screens [13]. They have also been used to sample *G. austeni* a tsetse fly species from the morsitans group (as was the case of in the eradication of *G. austeni* in Zanzibar with sterile insect technique, SIT) [13, 15, 16]. Further, some studies have used traps and targets covered with sticky material to assess the efficacy of these as landing devices [17, 18].

The ability of a sampling tool to catch tsetse flies particularly at low density is important as it could give information on residual populations and lead to appropriate action for planning and monitoring tsetse fly control interventions. A previous study showed that the small targets comprising of black and netting panels (each 0.25 × 0.25 m) caught over twice more *G. f. fuscipes* than the biconical trap [9]. Therefore, in this study we applied a sticky film to the small target having a black and blue panel without any nettings, and compared its performance as a sampling tool to the biconical trap which is commonly used for sampling *G*. *f. fuscipes* and other riverine tsetse flies. We also assessed the influence of colour on the landing response of male and female *G. f. fuscipes* on the sticky small target*.*

## Methods

### Study area

**Fig. 1:**
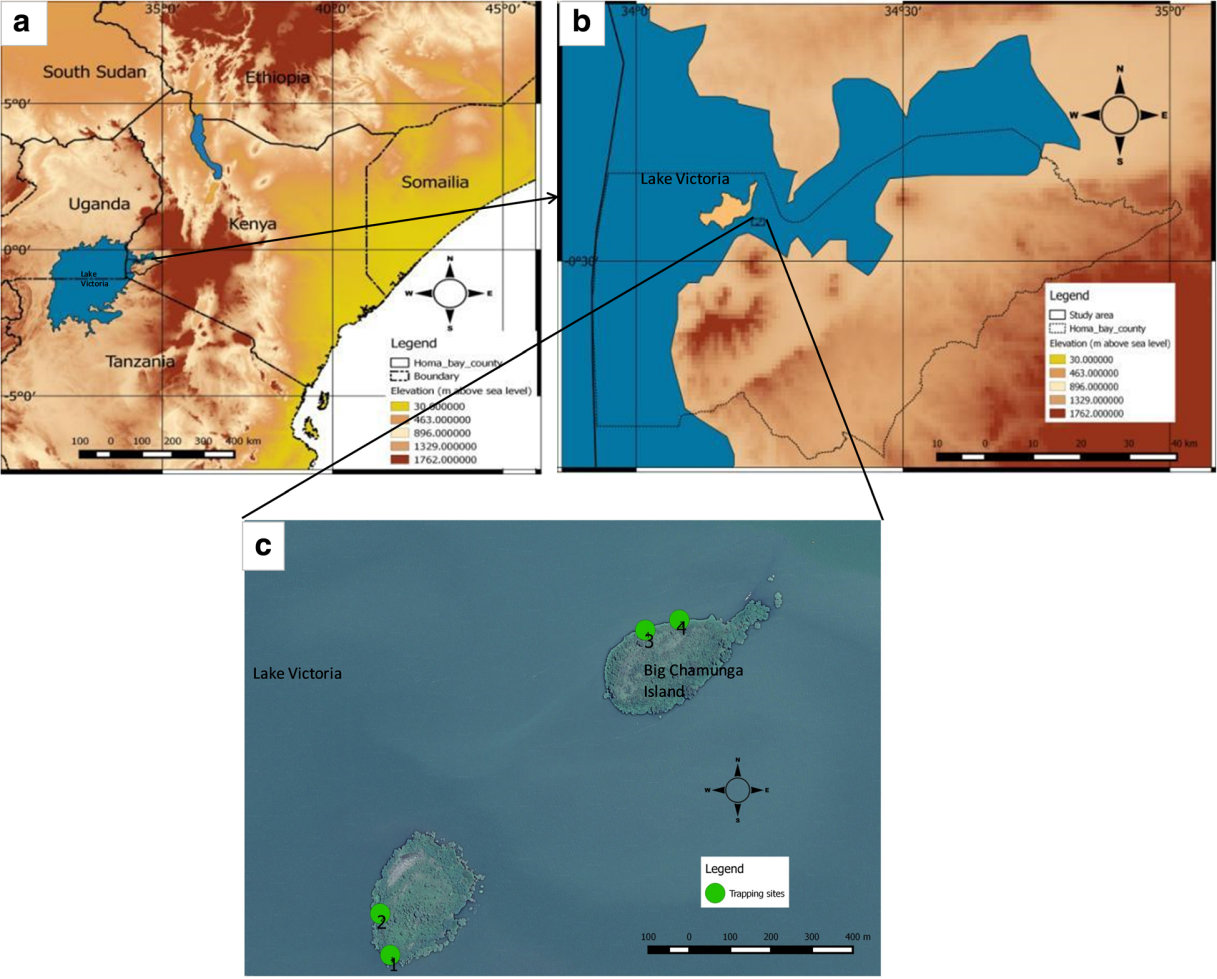
**a** Study area in western Kenya. **b** Location of study area in Homa Bay County. **c** Trapping sites on Big and Small Chamaunga Islands

The study was undertaken in the month of February 2017 on Big and Small Chamaunga Islands on Lake Victoria in western Kenya (Fig. 1). On these islands, *G. f. fuscipes* is the only tsetse fly species found and mainly inhabits the area along the lake shore [4, 9, 19, 20]. Study site selection was based on the fact that *G. f. fuscipes* populations on these islands were well documented and high numbers of flies are present for meaningful experimentation [4, 21]. These islands are not inhabited by humans and vegetation on the lake shore consists of a mixture of fresh water mangroves (*Aeschynomene eraphyroxylon*), tropical hydrangea (*Dombeya* spp*.*) and tickberry (*Lantana camara*). Monitor lizards (*Varanus niloticus*) are the main hosts for tsetse fly populations in the area [4].

### Sampling tools

**Fig. 2:**
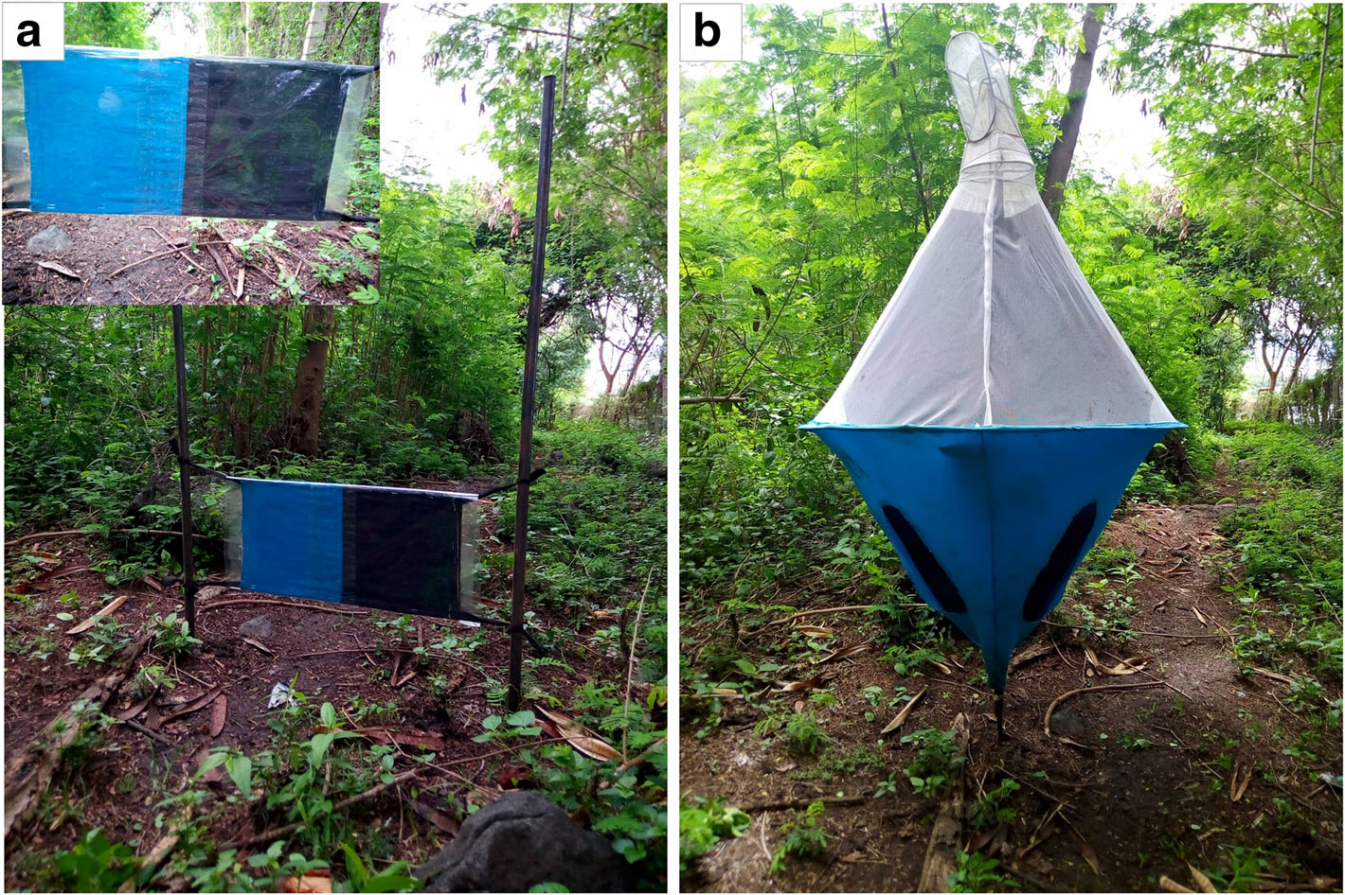
Sampling tools. **a** Sticky small target. **b** Biconical trap

Tsetse flies were caught using either biconical traps [22] or sticky small targets [9, 23]. The small target comprised of blue and black panels made from cotton cloth each 0.25 × 0.25 m in size and thus making it 0.25 × 0.50 m in dimension. A board 0.25 × 0.50 m in dimension of plywood was placed in between two small targets and fastened using staples. The board with the fastened targets was then covered with a transparent sticky film (Luminos 4 adhesive rolls-ungridded: Rentokil Initial supplies, Liverpool, UK) to make sticky small targets (Fig. 2). The sticky material on the target was not changed over the experimental period. Both sampling tools were not baited with any odour. The biconical traps had a radius of 0.40 m at its widest point, and a height of 1.30 m.

### Study design, sample collection and analyses

Field experiments with the traps and sticky small targets were undertaken for 4 h between 08:00 and 12:00 h local time when *G. f. fuscipes* are most active [3, 20], after which the flies collected in the non-return cage of the biconical trap and those on the sticky small target were sexed and counted. On the sticky small target, flies that landed on the blue or black panels were carefully removed using forceps and placed in separate storage containers colour-coded according to the part of the target that they landed on. The experiments were conducted over a period of 8 days at 4 sites. Sites 1 and 2 were on Small Chamaunga and sites 3 and 4 on Big Chamaunga (Fig. 1c). On the first and second day, experiments were carried out at two sites and from the third to eighth day, the experiments were undertaken at all four sites.

The overall tsetse fly catches from biconical traps and the sticky small targets were compared using 14 replicates of randomised block design experiments, each comprising of two adjacent days at a site as different blocks [24]. The sampling tools as treatments were randomly allocated to the days within the blocks and sites were at least more than 100 m apart [3]. A nested survey in the randomised block design was used to collect data on the landing response of female and male *G. f. fuscipes* on the blue or black panel of the sticky small target according to the method developed by Vale [25].

All statistical analyses were carried out using R version 3.2.5 [26]. Tsetse fly catches from the sampling tools were modeled using a negative binomial regression to determine the catch index of the sticky small target with the biconical trap as a reference while taking into account the block and day of study. The effect display (for detransformed means) of treatments in the negative binomial model was obtained using the ‘*effects*’ package in R [27]. Associations were tested using Fisher’s exact test. A *Z*-test was used to test for difference in proportions. Fly catches on the sticky small targets were further analysed using a multiple logistic regression to measure the association between the sex ratios of flies that landed on the blue or black panels while accounting for trap site and the day of the study. All estimates reported are accompanied by 95% confidence intervals and *P*-values less than 0.05 were considered statistically significant. All maps were created using QGIS version 2.10.1-Pisa.

## Results

### Characteristics of fly catches

A total of 441 flies were collected, 58 (13%; 95% CI: 10–16%) were caught in biconical traps while 383 (87%; 95% CI: 84–90%) were caught by the sticky small targets. The overall catches for the females and males were 290 (66%; 95% CI: 61–70%) and 151 (34%; 95% CI: 30–39%), respectively. The catches for the females in the biconical traps were 24 (41%; 95% CI: 28–54%) and those for the males were 34 (59%; 95% CI: 45–72%). The flies caught on the sticky small targets comprised of 266 (69%; 95% CI: 65–74%) females and 117 (31%; 95% CI: 26–35%) males. Overall, there was an association between fly sex and site of collection (Fisher’s exact test: *P* < 0.016, OR = 0.46, 95% CI: 0.25–0.83). The total number of females caught at all four sites was higher than those of males (Table 1). The flies caught on Small and Big Chamaunga were 209 (47%; 95% CI: 42–52%) and 232 (53%; 95% CI: 48–57%) respectively and their proportions were not significantly different (*Z*-test, *Z* = -1.55, *P* = 0.121).

**Table 1:** Fly catches at the four sites according to sex of *G. f. fuscipes*

Island	Site no	Female		Male		Total	
		Number	% (95% CI)	Number	% (95% CI)	Number	% (95% CI)
Small Chamaunga	1	97	70 (62–78)	42	30 (22–38)	139	32 (27–36)
	2	36	51 (39–63)	34	49 (37–61)	70	16 (12–19)
Big Chamaunga	3	55	61 (51–71)	35	39 (29–49)	90	20 (17–24)
	4	102	72 (64–79)	40	28 (21–36)	142	32 (28–36)
	Total	290	66 (61–70)	151	34 (30–39)	441	100

*Abbreviation*: *CI* confidence interval

### Comparison of sampling tools

Overall, the sticky small targets caught more *G. f. fuscipes* than the biconical traps (Table 2). Detransformed means were 3.2 (95% CI: 2.3–4.4) for the biconical trap compared to 23.3 (95% CI: 20.6–26.4) for the sticky small target (Table 2). The negative binomial regression showed that the sticky small target significantly caught 7.2 (95% CI: 5.3–10.1) times more flies (*Z* = 12.226, *P* < 0.0001) than the biconical trap after taking into account the variation due to the day of the experiment and the block. While according to sex, the sticky small target significantly caught 13.5 and 3.6 times more females and males, respectively compared to the biconical trap (*Z* = 9.551, *P* < 0.0001 and *Z* = 5.978, *P* < 0.0001, respectively).

**Table 2:** Means and indices of catches obtained from negative binomial model for female and male *G. f. fuscipes*

Treatment	Females			Males			Overall		
	Catch (95% CI)	Catch index (95% CI)	*P-*value	Catch (95% CI)	Catch index (95% CI)	*P-*value	Catch (95% CI)	Catch index (95% CI)	*P -* value
Biconical trap (control)	1.1 (0.6–1.9)	1		1.9 (1.2–3.1)	1		3.2 (2.3–4.4)	1	
Sticky small target	15.1 (12.8–17.8)	13.5 (8.2–24.4)	< 0.0001	6.9 (5.2–9.1)	3.6 (2.4–5.6)	< 0.0001	23.3 (20.6–26.4)	7.2 (5.3–10.1)	< 0.0001

*Abbreviation*: *CI* confidence interval

### Landing responses

Overall, from 383 flies that landed on the sticky small targets, 169 (44%; 95% CI: 39–49%) landed on the blue panel while 214 (56%; 95% CI: 51–61%) landed on the black panel. Overall, 108 (41%; 95% CI: 35–47%) female flies landed on the blue panel and while 158 (59%; 95% CI: 53–65%) landed on the black panel. For male flies, 61 (52%, 95% CI: 43–61%) landed on the blue panel while 56 (48%, 95% CI: 39–57%) landed on the black panel. From all the flies that landed on the blue panel 64% (95% CI: 56–71%) were female and 36% (95% CI: 28–43%) were male. Of the flies that landed on the black panel 73% (95% CI: 68–80%) were female while 26% (95% CI: 20–32%) were male. There was a statistically significant association between the proportion of flies that landed on the blue or black panels with sex of the fly (Fisher’s exact test, *P* < 0.044; OR: 1.59; 95% CI: 1.02–2.47). A multiple logistic regression showed that females had a 1.71 significant increase in the likelihood of landing on the black compared to the blue panel of the sticky small target than males and the increase could be as low as 1.07 and as high as 2.74 at 95% confidence interval while holding the trap site and day of study constant (*Z* = 2.25, *P <* 0.025). The trap site and day of study did not show any significant effect on the landing response (Table 3).

**Table 3:** Association between landing response on the blue or black panel of sticky small target with sex of *G. f. fuscipes*, site and day of study

Explanatory variable, total (*n* = 383)	UOR (95% CI)	*P*-value	AOR (95% CI)	*P*-value
Sex				
Male	1	na	1	na
Female	1.59 (1.02–2.47)	0.04*	1.71 (1.07–2.74)	0.025*
Site				
1	1	na	1	na
2	0.47 (0.24–0.90)	0.023*	1.13 (0.36–3.58)	0.838
3	1.02 (0.57–1.83)	0.931	1.30 (0.53–3.20)	0.570
4	1.11 (0.68–1.83)	0.664	1.13 (0.63–2.01)	0.679
Day of study				
1	1	na	1	na
2	0.33 (0.11–0.95)	0.040*	0.30 (0.08–1.15)	0.079
3	0.92 (0.44–1.90)	0.822	0.84 (0.37–1.92)	0.684
4	1.13 (0.48–2.66)	0.780	0.98 (0.39–2.43)	0.964
5	1.85 (0.86–4.01)	0.117	1.90 (0.82–4.41)	0.135
6	0.53 (0.20–1.44)	0.215	0.48 (0.16–1.41)	0.135
7	0.97 (0.43–2.18)	0.949	0.82 (0.34–1.98)	0.653
8	1.23 (0.51–2.95)	0.648	1	na

*Abbreviations*: *UOR* unadjusted odds ratio, *AOR* adjusted odds ratio, *CI* confidence interval, *na* not applicable

*Statistical significance at *P* < 0.05

## Discussion

The present study revealed that sticky small targets are more efficient sampling tools for *G. f. fuscipes* than the biconical traps which are the most commonly used sampling tools for the species. In another study, blue/black targets that were 1.0 × 1.5 m in dimension covered with sticky film caught an average of about 4 to 6 times more *G. f. fuscipes* than biconical traps [28]. The results from our study are comparable with the sticky small targets of dimensions 0.25 × 0.50 m catching 7 times more flies than the biconical traps suggesting that they could be an alternative and more effective sampling tool for *G. f. fuscipes.* Furthermore, the higher proportion of females compared to males caught on the sticky small target is more representative of the sex ratio in the population [13] which also makes this sampling tool more useful for ecological studies. Previous versions of a sticky trap for tsetse flies were comprised of a coloured metal, wooden or cloth screen coated with sticky substance [13]. These have not been popular with workers due to the difficulty in collecting and handling the flies and the poor condition of the flies in subsequent processing such as counting and dissections [13]. As opposed to the sticky substances coated on the screens of the earlier sticky traps, the transparent sticky film used in this study did not present any difficulties in collecting, handling and counting the flies. Additionally, all the flies collected on the sticky small target were easily distinguished by sex, an indication that the samples were in good condition. This is consistent with observations from other studies that used the transparent sticky film on targets to sample other tsetse fly species [23]. However, further studies are needed to assess whether samples collected from sticky small targets could be used for dissections and molecular biological studies. Additionally, with the current knowledge, sticky small targets could do particularly well in circumstances requiring highly efficient sampling for *G. f. fuscipes* such as determining its distribution limits, mark-release-recapture experiments and monitoring of residual populations. All these are important in the planning and implementation of tsetse fly control interventions.

Studies on the cost effectiveness of small targets indicate that they use 1/24th of the material in the biconical trap and are much easier to deploy [9, 29]; thus, their use in control of *G. f. fuscipes* could substantially increase field cost-effectiveness by a factor of 10 [9, 30]. In our study we recommend the use of the small targets covered with sticky material for sampling as they significantly caught more *G. f. fuscipes* than biconical traps. Although further cost-effectiveness studies need to be undertaken, the additional costs of the sticky material and its advantages as seen in the current study to sample fly populations and carry out ecological studies could justify the slight increase in costs particularly when monitoring tsetse fly populations during control interventions.

In order to address variations, other than those due to the variables of interest, we used randomised block design experiments, where treatments were randomly assigned to experimental units in a block. This randomisation ensured that confounds were controlled for. Further, residual confounding was addressed during statistical analysis by accounting for the block and day the experiments were undertaken. Additionally, for assessment of the influence of the colour on the landing response of *G. f. fuscipes* according to sex, confounds were addressed during analysis by accounting for the site and day of the study in the multiple logistic regression.

Our results are also consistent with earlier studies which showed that a larger proportion of *G. f. fuscipes* land on the black panel of the target than the blue panel [28]. Similar landing behaviour have also been reported for species belonging to the morsitans group such as *G. m. morsitans* and *G. pallidipes* [25, 31]*.* The observation that females show a preference to land on the black than on the blue panel supports earlier practices of impregnating the black panel of the target with insecticide to kill females [25]. As an alternative to insecticides, bio-control agents such as entomopathogenic fungi have been shown to be efficacious against *G. f. fuscipes* and can be horizontally transmitted from infected to non-infected tsetse flies [32, 33]. As indicated in our results, the most likely point of first contact for male flies is the blue panel of the small target, thus it could be impregnated with a bio-control agent. In this case, the male flies having a preference to land on the blue than black panel of the small target could be contaminated and serve as carriers to deliver the bio-control agent to females during mating. With the male fly mating more than once in its lifetime; the bio-control agent would be transferred to each female it mates with [8]. Likewise, though to a lesser extent, unmated females that are contaminated with the agent could also transfer it to other males. The bio-control agent impregnated small targets can be used in an integrated manner either concurrently or sequentially with those treated with insecticide. However, studies to determine the feasibility and cost effectiveness of such integrated methods would be required. Notwithstanding, exploiting landing preference of male *G. f. fuscipes* on the blue panel in the manner suggested could provide an opportunity for more target based integrated riverine tsetse fly control strategies.

## Conclusion

Sticky small targets significantly caught more flies than biconical traps suggesting that they are more efficient sampling tools for *G. f. fuscipes.* Our study also showed male *G. f. fuscipes* preferred to first land on the blue part of the small target; a behaviour that could be exploited for disseminating biological control agents in populations for tsetse control.

## Abbreviations

CI: confidence interval
HAT: human African trypanosomiasis
gHAT: Gambian human African trypanosomiasis

## References

[1] WHO (2014). Report of the first WHO stakeholders meeting on gambiense human African trypanosomiasis elimination.

[2] WHO (2015). Report of the first WHO stakeholders meeting on rhodesiense human African trypanosomiasis elimination.

[3] Omolo MO, Hassanali A, Mpiana S, Esterhuizen J, Lindh J, Lehane MJ (2009). Prospects for developing odour baits to control *Glossina fuscipes* spp., the major vector of human African trypanosomiasis. PLoS Negl Trop Dis.

[4] Tirados I, Esterhuizen J, Kovacic V, Mangwiro TNC, Vale GA, Hastings I (2015). Tsetse control and Gambian sleeping sickness; implications for control strategy. PLoS Negl Trop Dis.

[5] Welburn SC, Molyneux DH, Maudlin I (2016). Beyond tsetse - implications for research and control of human African trypanosomiasis epidemics. Trends Parasitol.

[6] Sudarshi D, Lawrence S, Pickrell WO, Eligar V, Walters R, Quaderi S (2014). Human African trypanosomiasis presenting at least 29 years after infection - what can this teach us about the pathogenesis and control of this neglected tropical disease?. PLoS Negl Trop Dis.

[7] Checchi F, Filipe JAN, Haydon DT, Chandramohan D, Chappuis F (2008). Estimates of the duration of the early and late stage of gambiense sleeping sickness. BMC Infect Dis.

[8] Vreysen MJ, Seck MT, Sall B, Bouyer J (2013). Tsetse flies: their biology and control using area-wide integrated pest management approaches. J Invertebr Pathol.

[9] Lindh JM, Torr SJ, Vale GA, Lehane MJ (2009). Improving the cost-effectiveness of artificial visual baits for controlling the tsetse fly *Glossina fuscipes fuscipes*. PLoS Negl Trop Dis.

[10] Torr S, Chamisa A, Vale G, Lehane M, Lindh JM (2011). Responses of tsetse flies, *Glossina morsitans morsitans* and *Glossina pallidipes*, to baits of various size. Med Vet Entomol.

[11] Rayaisse JB, Esterhuizen J, Tirados I, Kaba D, Salou E, Vale GA, et al. Towards an optimal design of target for tsetse control: comparisons of novel targets for the control of Palpalis group tsetse in West Africa. 2011;5:1–8. 10.1371/journal.pntd.0001332 PMC3176748 21949896

[12] WHO. Strategic review of traps and targets for tsetse and African trypanosomiasis control. World Heal Rep. 2004:1–58.

[13] Leak S, Ejigu D, Vreysen M (2008). Collection of entomological baseline data for tsetse area-wide integrated pest management programmes. FAO/IAEA.

[14] Challier A, Gouteux JP (1980). Ecology and epidemiological importance of *Glossina palpalis* in the Ivory Coast forest zone. Insect Sci Its Appl.

[15] Vreysen MJB, Saleh KM, Ali MY, Abdulla AM, Zhu Z-R, Juma KG (2000). *Glossina austeni* (Diptera: Glossinidae) eradicated on the Island of Unguja, Zanzibar, using the sterile insect technique. J Econ Entomol.

[16] Vreysen MJB, Zhu ZR, Saleh KM (1998). Field responses of *Glossina austeni* to sticky panels on Unguja Island, Zanzibar. Med Vet Entomol.

[17] Rayaisse JB, Kröber T, McMullin A, Solano P, Mihok S, Guerin PM (2012). Standardizing visual control devices for tsetse flies: West African species *Glossina tachinoides*, *G. palpalis gambiensis* and *G. morsitans submorsitans*. PLoS Negl Trop Dis.

[18] Mramba F, Oloo F, Byamungu M, Krober T, McMullin A, Mihok S (2013). Standardizing visual control devices for tsetse flies: East African species *Glossina swynnertoni*. PLoS Negl Trop Dis.

[19] Mwangelwa MI. Ecology and vectorial capacity of *Glossina fuscipes fuscipes* Newstead, 1910 on Rusinga Island and along the shores of Lake Victoria, Kenya. PhDThesis. The University of Zambia, Lusaka, Zambia; 1990. http://dspace.unza.zm:8080/xmlui/handle/123456789/1904?show=full

[20] Mohamed-Ahmed M, Odulaja A (1997). Diel activity patterns and host preferences of *Glossina fuscipes fuscipes* (Diptera: Glossinidae) along the shores of Lake Victoria, Kenya. Bull Entomol Res.

[21] Mbewe NJ, Saini RK, Torto B, Irungu J, Yusuf AA, Pirk C (2018). Effects of vector control on the population structure of tsetse (*Glossina fuscipes fuscipes* ) in western Kenya. Acta Trop.

[22] Challier A, Laveissiere C (1973). A new trap for catching flies (Glossina: Diptera, Muscidae): description and field trials. Cah ORSTOM Entomol Med Parasitol.

[23] Rayaisse J, Salou E, Courtin F, Yoni W, Barry I, Dofini F (2015). Baited-boats: an innovative way to control riverine tsetse, vectors of sleeping sickness in West Africa. Parasit Vectors.

[24] Torr S, Mangwiro T, Hall D (2011). Shoo fly, don’t bother me! Efficacy of traditional methods of protecting cattle from tsetse. Med Vet Entomol.

[25] Vale GA. Visual responses of tsetse flies (Diptera: Glossinidae) to odour-baited targets. Bull Entomol Res. 1993:277–89.

[26] R Core Team (2016). R: A language and environment for statistical computing.

[27] Fox J (2003). Effect displays in R for generalised linear models. J Stat. Softw.

[28] Oloo F, Sciarretta A, Mohamed-Ahmed MM, Kröber T, McMullin A, Mihok S (2014). Standardizing visual control devices for tsetse flies: East African species *Glossina fuscipes fuscipes* and *Glossina tachinoides*. PLoS Negl Trop Dis.

[29] Esterhuizen J, Rayaisse JB, Tirados I, Mpiana S, Solano P, Vale GA (2011). Improving the cost-effectiveness of visual devices for the control of riverine tsetse flies, the major vectors of human African trypanosomiasis. PLoS Negl Trop Dis.

[30] Shaw APM, Torr SJ, Waiswa C, Cecchi G, Wint GRW, Mattioli RC (2013). Estimating the costs of tsetse control options: An example for Uganda. Prev Vet Med.

[31] Vale GA (1993). Development of baits for tsetse flies (Diptera: Glossinidae) in Zimbabwe. J Med Entomol.

[32] Maniania NK, Ekesi S (2012). The use of entomopathogenic fungi in the control of tsetse flies. J Invertebr Pathol.

[33] Maniania NK, Ekesi S, Odulaja A, Okech MA, Nadel DJ (2006). Prospects of a fungus-contamination device for the control of tsetse fly *Glossina fuscipes fuscipes*. Biocontrol Sci Technol.

